# Case Studies in Molecular Network-Guided Marine Biodiscovery

**DOI:** 10.3390/md21070413

**Published:** 2023-07-20

**Authors:** Shamsunnahar Khushi, Angela A. Salim, Robert J. Capon

**Affiliations:** Institute for Molecular Bioscience, The University of Queensland, St Lucia, QLD 4072, Australia; khusi@pharm.ku.ac.bd (S.K.); a.salim@uq.edu.au (A.A.S.)

**Keywords:** global natural products social molecular networking, GNPS, marine natural products

## Abstract

In reviewing a selection of recent case studies from our laboratory, we revealed some lessons learned and benefits accrued from the application of mass spectrometry (MS/MS) molecular networking in the field of marine sponge natural products. Molecular networking proved pivotal to our discovery of many new natural products and even new classes of natural product, some of which were opaque to alternate dereplication and prioritization strategies. Case studies included the discovery of: (i) trachycladindoles, an exceptionally rare class of bioactive indole alkaloid previously only known from a single southern Australia sample of *Trachycladus laevispirulifer*; (ii) dysidealactams, an unprecedented class of sesquiterpene glycinyl-lactam and glycinyl-imide from a *Dysidea* sp., a sponge genera often discounted as having been exhaustively studied; (iii) cacolides, an unprecedented family of sesterterpene α-methyl-γ-hydroxybutenolides from a *Cacospongia* sp., all too easily mischaracterized and deprioritized during dereplication as a well-known class of sponge sesterterpene tetronic acids; and (iv) thorectandrins, a new class of indole alkaloid which revealed unexpected insights into the chemical and biological properties of the aplysinopsins, one of the earliest and more extensively reported class of sponge natural products.

## 1. Introduction

Marine sponges are a well-established source of structurally diverse natural products, many with no biosynthetic counterparts in other life forms, whether marine or terrestrial, plant, animal, or microbial [1]. Over a period spanning several decades, our laboratory investigated the chemistry of many southern Australian marine sponges, leading to the discovery of numerous new natural products. Notwithstanding past successes, in recent years the field of marine sponge natural products research experienced a slow-down in reports of new natural products. There are several factors that might contribute to this decline, a key one of which is the challenge of dereplication–where an inability to rapidly and cost effectively detect and prioritize new over known and rare over common natural products results in the rediscovery of known chemistry and an overall decrease in productivity. Over the years, natural products researchers relied on a number of dereplication strategies with varying levels of success. For example, in the early days, thin layer chromatography (TLC) was used extensively, evolving over time to normal and then reversed phase high-performance liquid chromatography (HPLC) and, in time, to ultra-high performance liquid chromatography (UPLC), with columns featuring ever smaller particle sizes and resolving power. Chromatographic methods also benefited from an evolution in detectors ranging from refractive index (RI) to single wavelength ultra-violet (UV-vis), diode array (DAD), evaporative light scattering (ELS), and electro-spray mass spectrometry (ESI-MS). In time, mass spectrometric detectors advanced in both sensitivity and accuracy (UPLC-DAD-QTOF), with data analysis further enabled by such techniques as single ion extraction (SIE) and tandem mass spectrometry (MS^n^). With dereplication based on sophisticated mass spectrometric analysis generating an almost bewildering wealth of data, it was a logical (albeit challenging) next step to develop innovative computational tools to process and visualise these data to more easily and rapidly interrogate very large data sets incorporating many 1000s even 10,000s of compounds. Importantly, these mass spectrometric approaches required only a few μg of crude extract (or fractions or pure compounds) with individual data acquisitions taking only a few minutes and at minimal cost. In recent years, one of the more popular mass-spectrometry-based dereplication methodologies is that of Global Natural Products Social (GNPS) molecular networking [2].

Since its emergence in 2016 [3], GNPS molecular networking has been widely used by marine natural products researchers; for instance, in 2017 Crusemann et al. applied GNPS molecular networking to prioritize 146 marine *Salinispora* and *Streptomyces* strains, which revealed 15 natural products families and enabled identification of new analogues including metabolite production patterns and efficient growth and extraction conditions [4]; in 2020 Mangoni et al. applied GNPS to discover a new proline-rich cyclic heptapeptide stylissamide L from a well-studied marine sponge *Stylissa caribica* [5], while Keyzers et al. applied GNPS molecular networking to isolate new rubrolide analogues from the New Zealand marine tunicate *Synoicum kuranui* [6]. Our own laboratory also used GNPS on multiple occasions, including during investigations into mullet fish gut-derived fungi which lead to: new lipodepsipeptide scopularides from *Scopulariopsis* spp. CMB-F458 and CMB-F115, and *Beauveria* sp. CMB-F585 [7]; new P-glycoprotein inhibitory phenyl propanoid piperazine alkaloid chrysosporazines from *Aspergillus* sp. CMB-F661, *Spiromastix* sp. CMB-F455, *Chrysosporium* sp. CMB-F214, and *Chrysosporium* sp. CMB-F294 [8,9,10]; and new polyketide amaurones from *Amauroascus* sp. CMB-F713 [11].

In this perspective we seek to demonstrate through a selection of case studies how we successfully applied GNPS molecular networking to breathe new life into a library of 960 southern Australian marine extracts (predominantly sponges). This library was the focus of extensive study in our group for over 30 years, but in recent years it was largely sidelined due to a perception it was a near-exhausted resource. As demonstrated below, the acquisition and analysis of a GNPS molecular network on this library revealed a wealth of new opportunities, with an investigation of just a few of these newly revealed opportunities prompting discoveries that might otherwise have been overlooked. These include, for example, an alternate source and new exemplars of the exceptionally rare class of highly selective kinase inhibitory alkaloid, trachycladindoles from *Geodia* sp. (CMB-001063) [12]; an unprecedented class of sesquiterpene glycinyl lactam and imide, dysidealactams from *Dysidea* sp. (CMB-01171) [13]; a new class of sesterterpene featuring an unprecedented α-methyl-γ-hydroxybutenolide, cacolides from *Cacospongia* sp. [14]; and a new class of indole alkaloid, thorectandrin A from *Thorectandra choanoides* (CMB-01889) [15].

## 2. Acquiring a GNPS Molecular Network of a Marine Library

### 2.1. Preparing a Marine Extract Library

Over a period of 35+ years our laboratory assembled a marine natural products biodiscovery resource consisting of >3000 marine invertebrates and macroalgae sourced from locations across southern Australia and Antarctica. This marine collection proved to be a valuable resource for marine natural products research, informed by an array of bioassays across human and animal health, and crop protection (i.e., targeting such indications as anti-infective and neurodegenerative diseases, cancer, pain, etc.), as well as unusual chemical parameters detected using an evolving array of dereplication technologies (i.e., TLC, HPLC-RI-UV, HPLC-DAD, HPLC-ESI(±)MS, and NMR spectroscopy). In total ~15% of the library was subjected to detailed chemical analysis, resulting in the discovery of many hundreds of novel marine natural products, some with promising (even commercially significant) biological properties. Notwithstanding this success, this left ~85% of the collection designated of lesser (no?) interest. Given the propensity of marine organisms to produce chemical defences this deprioritization was likely an artifact of less than optimal dereplication methods. We were therefore intrigued to observe the emergence of GNPS molecular networking, rationalising that this methodology may hold the means to better assess and hopefully reprioritize some of our stranded marine extracts. Once we had secured access to a suitable UPLC-QTOF mass spectrometer we elected to carry out a pilot study to better understand this dereplication paradigm, applying GNPS molecular networking to a selection of southern Australian marine extracts.

A library of 960 marine extracts (predominantly sponges) was chosen, as it could be conveniently accommodated in 10 × 96 well plates. Our selection also included several marine sponge extracts that we previously studied and for which we had knowledge of their natural products (Table 1). To support dereplication of these known natural product classes we also cross-correlated with a separate library of 95 pure authentic marine natural products. In total our library of 960 extracts were derived from 409 sponges with taxonomy to at least the level of genus (Table 2), along with a further 551 taxonomically unidentified samples, comprising 384 sponges, 49 tunicates, and 118 macroalgae. All samples were collected over the last 35 years by either intertidal (0–1 m), near shore SCUBA (5–20 m), or commercial trawling or scientific benthic sleds operations (20–500 m), from locations across the southern Australian coast, south to Antarctic waters. Soon after collection all samples were individually bagged and frozen (or placed on ice) for transport to the laboratory, where they were diced and stored (extracted) in sealed Nalgene bottles in 10% water/EtOH at −20 °C (for up to 35 years).

In order to carry out our GNPS analyses, fresh individual aliquots (7 mL) dispensed from the 10% water/EtOH extracts of 960 samples were concentrated to dryness in vacuo at 45 °C, after which they were partitioned between n-BuOH (2 mL) and water (2 mL). Aliquots (1 mL) of both the n-BuOH and water phases were transferred to deep 96-well plates to generate a set of stock extract plates, which were stored at –30 °C. Aliquots (100 μL) of the n-BuOH solubles were transferred to a second set of deep 96-well plates, after which they were dried under nitrogen gas at 40 °C and redissolved in DMSO (1 mL) to prepare a 10-fold dilution daughter plates to be used for GNPS analysis. All plates were stored at –30 °C in the dark until required.

### 2.2. Preparing a GNPS Molecular Network

**Figure 1:**
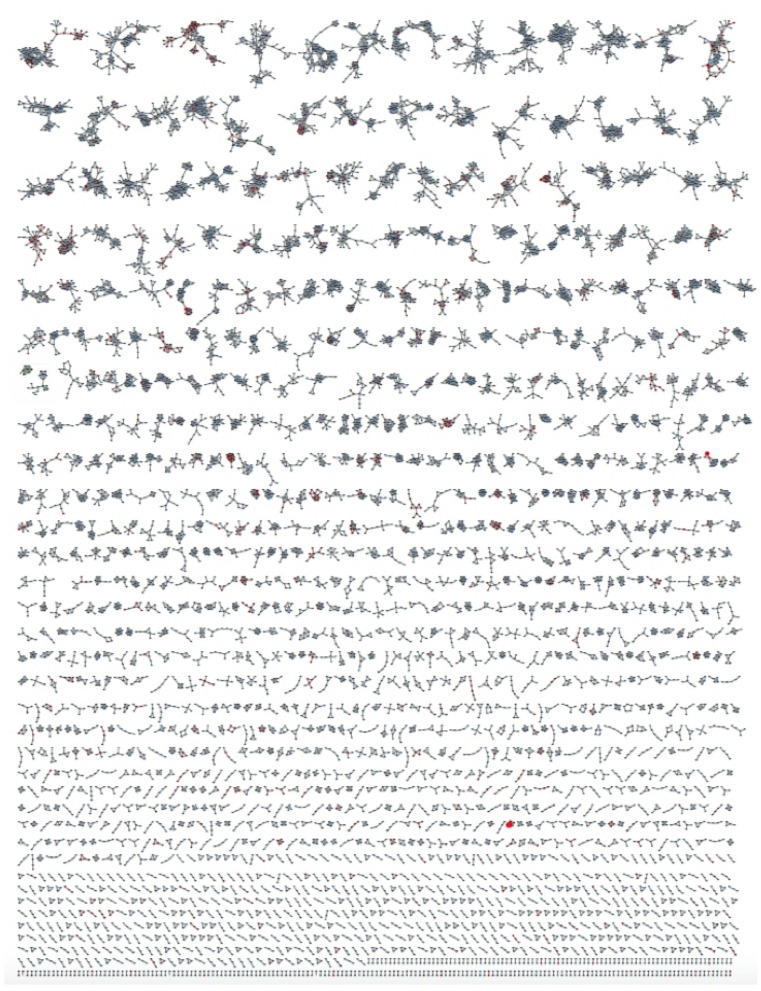
GNPS molecular network for 960 marine extracts (blue nodes) including marine sponge extracts containing known chemistry (red nodes), superimposed over 95 authentic pure natural product standards (green nodes). See the Supplementary Materials for a higher resolution image.

Aliquots (0.1 μL) of the daughter plates (960 extracts and 95 authentic pure marine natural products) were analysed on an Agilent 6545 QTOF LC/MS equipped with an Agilent 1290 infinity II UHPLC system, utilising an Agilent SB-C8 1.8 μm, 2.1 × 50 mm column, with a 0.5 mL min^−1^ gradient elution over 4.5 min from 90% H_2_O/CH_3_CN to CH_3_CN, followed by isocratic elution with CH_3_CN for 1 min, with a constant isocratic 0.1% formic acid/CH_3_CN modifier. The UPLC-QTOF(+)MS/MS data acquired for all samples at a fixed collision energy of 40 eV were converted from Agilent MassHunter data files (.d) into mzXML file format, and transferred to the GNPS server (gnps.ucsd.edu). Molecular networking was performed using the GNPS data analysis workflow using the spectroscopic clustering algorithm, and a cosine score of 0.7 and a minimum of six matched peaks. The resulting spectroscopic networks were imported into Cytoscape version 3.5.1, where nodes represented parent *m/z* and edge thickness corresponded to cosine scores, for a network featuring ~43,000 nodes, and hundreds of clusters (Figure 1). Analysis of the GNPS cluster revealed the presence of new analogues of known chemical scaffolds, as well as unprecedented new classes of compounds.

## 3. Detecting New Analogues of Known Chemistry

**Figure 2:**
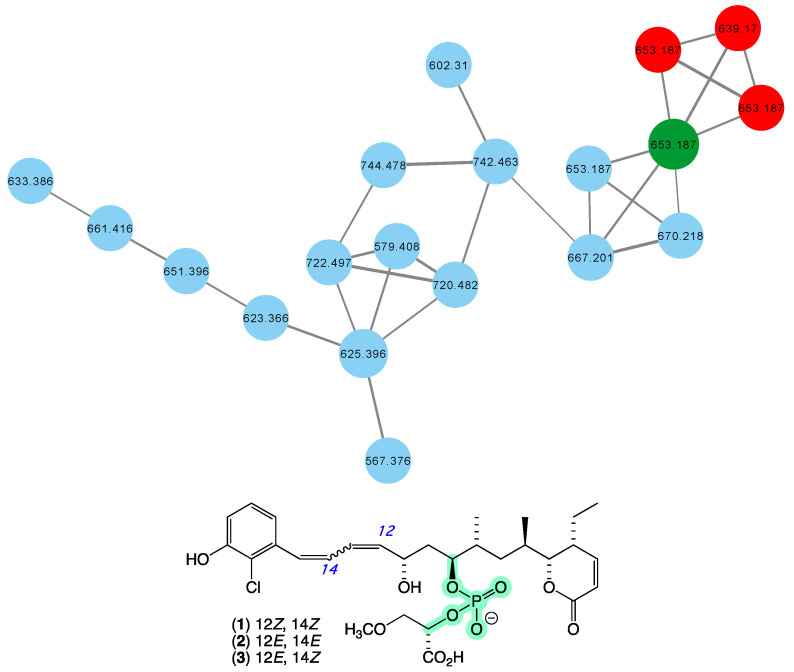
GNPS molecular family and structures for franklinolides. Highlights: authentic sample of **1** (dark green), franklinolides in the original CMB-01989 extract (red), potential new franklinolides in CMB-01451 and CMB-01518 extracts (blue), and the rare phosphodiester moiety in **1** (light green).

**Figure 3:**
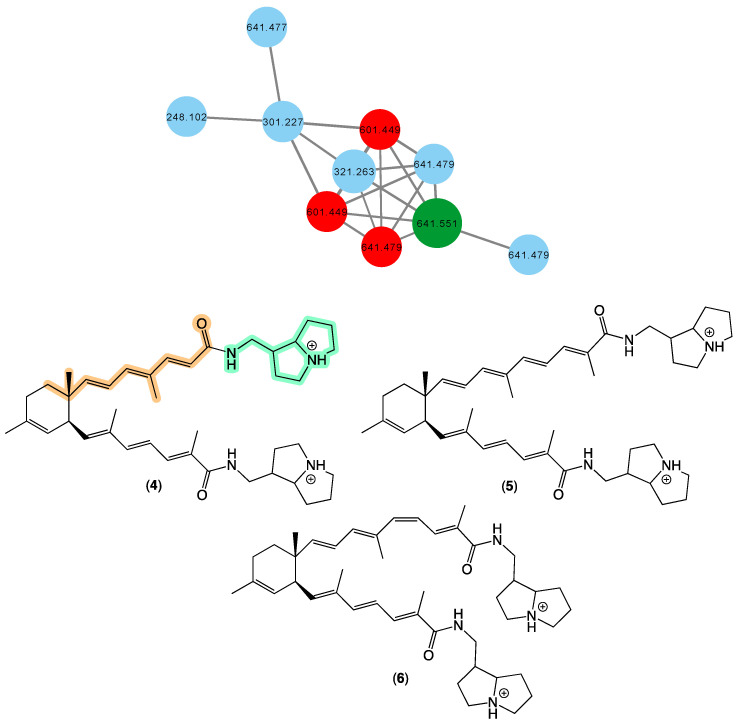
GNPS molecular family and structures for bistellettazines. Highlights: authentic sample of **4** (dark green), bistellettazines in CMB-01986 extract (red), potential new bistellettazines in the CMB-01021 extract (blue), and the pyrrolizidine (light green) and terpene (tan) sub-structures in **1**.

**Figure 4:**
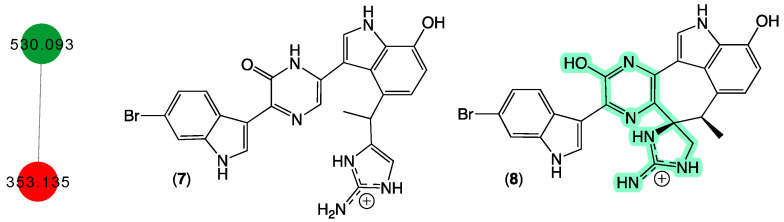
GNPS molecular family and structures for dragmacidins. Highlights: authentic **8** (dark green), **7** in CMB-02782 extract (red), and unusual chromaphore associated with **8** (light green).

**Figure 5:**
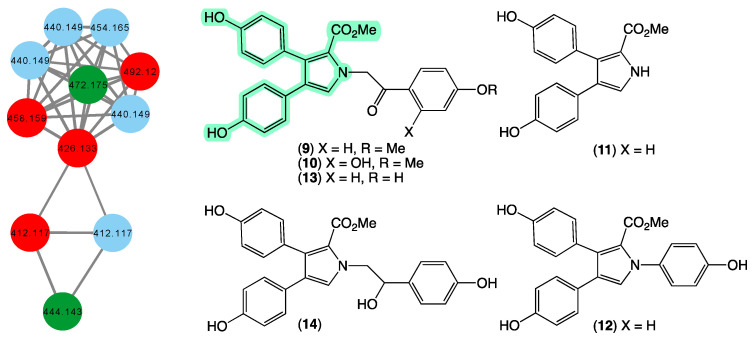
GNPS molecular family and structures for lamellarins. Highlights: authentic samples of **13** and the mono methyl ether of **9** (dark green), related lamellarins in the original CMB-01245 extract (red), new lamellarins in CMB-01311 (blue), and the pyrrole core common across **9**–**14** (light green).

**Figure 6:**
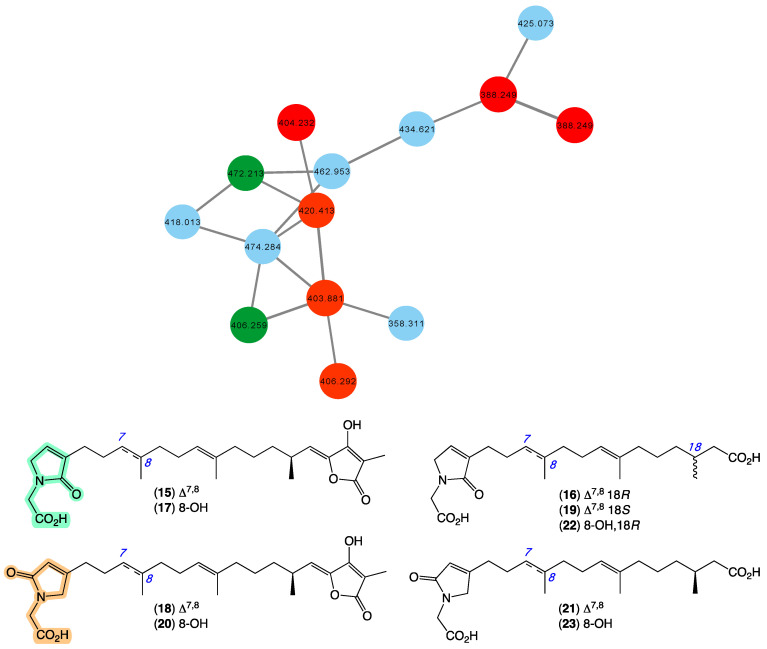
GNPS molecular family and structures for ircinialactams. Highlights: authentic samples of **15**–**16** (dark green), related ircinialactams in the original CMB-01064 extract (red), new lamellarins in CMB-01017 (blue), and the glycinyl-lactam regioisomeric sub-structures common across **9**–**14** (light green and tan).

**Figure 7:**
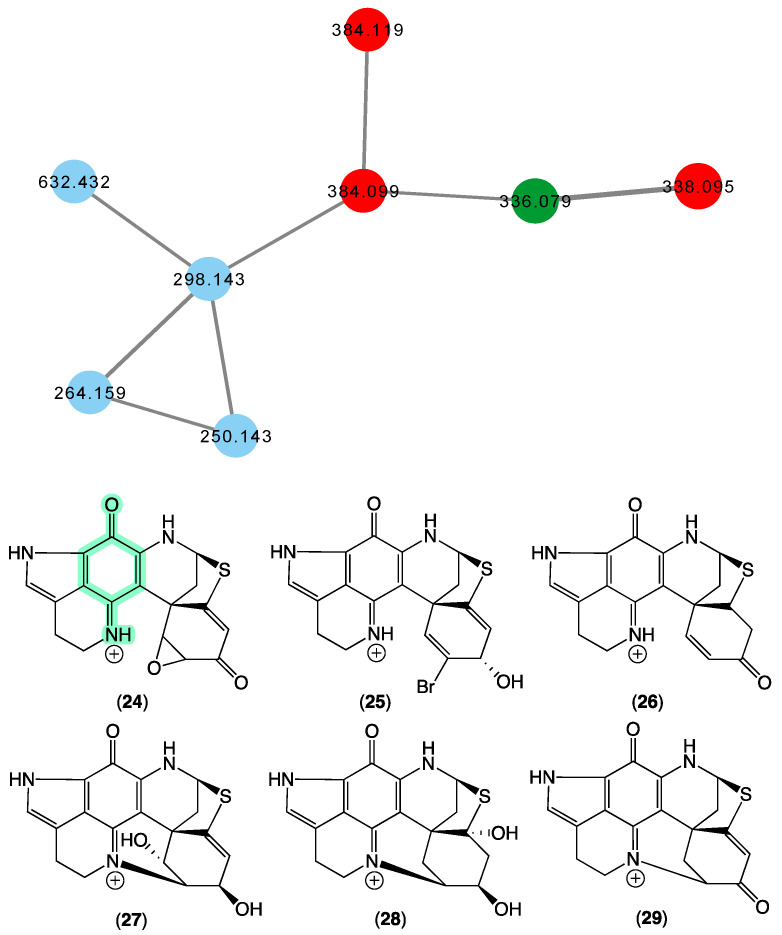
GNPS molecular family and structures for discorhabdins. Highlights: authentic sample of **29** (dark green), related discorhabdins in CMB-02720 and CMB-02523 (red), new discorhabdins in CMB-01879 (blue), and the pyrroloiminoquinone sub-structure common to **24**–**29** (light green).

**Figure 8:**
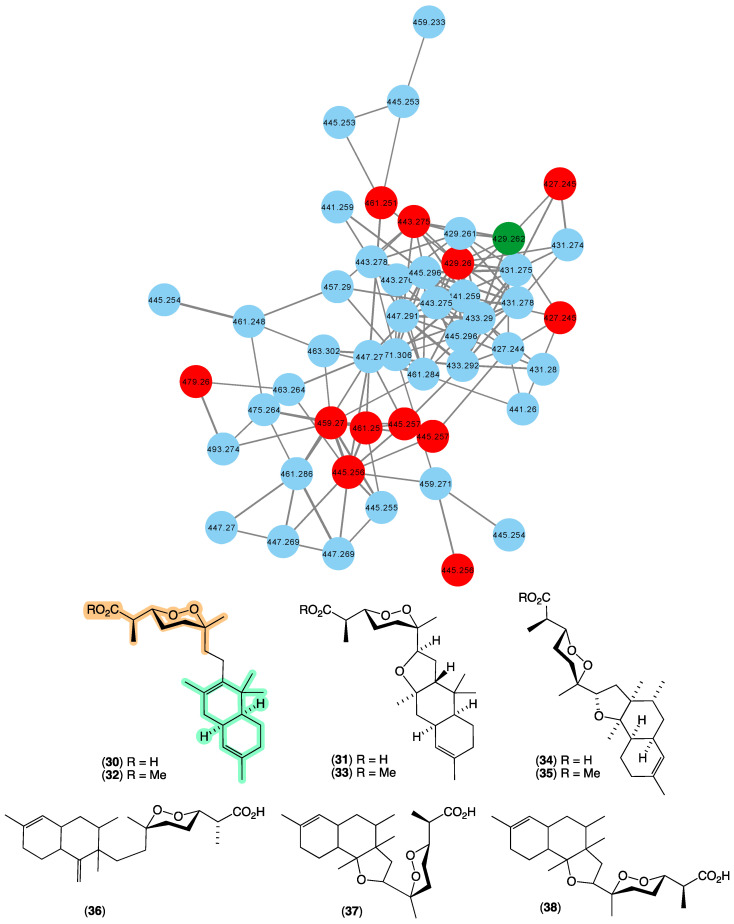
GNPS molecular family and structures for trunculins. Highlights: authentic sample of **30** (dark green), related trunculins in the original CMB-01738 extract (red), new trunculins in CMB-01001, CMB-01723 and CMB-01179 (blue), and the trunculin carbo/heterocyclic skeleton and cyclic peroxide sub-structure common across **30**–**38** (light green and tan).

Analysis of GNPS molecular networking (Figure 1) revealed several molecular families (Figures 2–8), where the pure authentic samples co-clustered with the original producing organisms, validating the use of GNPS to detect the presence of known compounds and their analogues in a large collection of extracts. Exemplars of these molecular families are discussed herein, with a brief introduction to their original isolations from the producing organisms and their biological activities.

### 3.1. Franklinolides

In 2010 [16], we reported franklinolides A–C (**1**–**3**) (Figure 2) from a marine sponge-complex (CMB-01989) consisting of a massive *Geodia* sp. thinly encrusted with a *Halichondria* sp., collected in 1995 during deep water (−105 m) scientific trawling operations in the Great Australian Bight. The franklinolides were the first reported natural occurrence of polyketide phosphodiesters, and inhibited the growth of a range of human carcinoma cells, including colon (HT-29), prostate (DU145), ovary (JAM and C180-13S), and lung (A549). With the exception of a 2016 re-isolation of franklinolide A (**1**) from a Caribbean sponge *Plakina jamaicensis* [27], no other members of this structure class have been reported, with a 2022 review on natural phosphate esters reinforced the rarity of natural phosphodiesters [28]. While traditional dereplication approaches failed to detect alternate sources of franklinolides in our marine library, the GNPS molecular network shown in Figure 2 revealed a family of nodes clustered around the authentic natural product franklinolide A (**1**) (dark green) and analogues in the original CMB-01989 extract (red), associated with new nodes (blue) present in two unidentified sponges (CMB-01451 and CMB-01518) collected in 1993 by SCUBA (−15 m) in Port Phillip Bay, Victoria. Based on this analysis, these latter sponges are worthy of more detailed chemical analysis, and are expected to provide access to new franklinolides.

### 3.2. Bistellettazines

In 2008 [17], we reported bistellettazines A–C (**4**–**6**) from a *Stelletta* sp. (CMB-01936) collected in 1995 during scientific trawling in the Great Australian Bight. The bistellettazines were then, and remain, the only reported examples of terpenyl-pyrrolizidine conjugates, and their biosynthesis was speculated to proceed via a Diels–Alder-like addition between two hypothetical polyenyl norsesquiterpene precursors. The GNPS molecular network shown in Figure 3 revealed a family of nodes clustered around the authentic natural product bistellettazine A (**4**) (dark green) and analogues in the original CMB-01986 extract (red), and new nodes (blue) associated with the unidentified sponge CMB-01021 collected in 1989 during scientific trawling operations in Bass Strait, Victoria. Based on this analysis, CMB-01021 is worthy of more detailed chemical analysis, and could provide access to new bistellettazines, including hypothetical biosynthetic precursor monomers.

### 3.3. Dragmacidins

In 1998 [18], we reported two new protein phosphatase inhibitors, dragmacidins D–E (**7**–**8**) from a *Spongosorites* sp. (CMB-01931) collected in 1991 during scientific trawling operations (−90 m) in the Great Australian Bight. Curiously, while both **7** and **8** were yellow pigments, **8** with its unique heterocyclic scaffold was an especially brilliant fluorescent yellow and proved tenacious at staining laboratory glassware. Although our CMB-01931 extract had been fully consumed in earlier studies, we were aware of another sponge sample in our collection that produced dragmacidins; CMB-02782 identified as a *Leiosella* sp. encrusted with a *Halichondria* sp. was collected in 1998 during scientific trawling operations in the Great Australian Bight. The GNPS molecular network shown in Figure 4 revealed a family of nodes clustered around the authentic natural product dragmacidin E (**8**) (dark green) and nodes associated with the CMB-01986 extract (red). Significantly, despite screening 960 specimens, we did not detect any other sponge extracts with nodes co-clustering with **8**, suggesting that this class of dragmacidin are quite rare.

### 3.4. Lamellarins

In 1994 [29], we reported on the first example of pyrrolo-alkaloid lamellarins from a marine sponge, namely lamellarins O–P (**9**–**10**) from a specimen of *Dendrilla cactos* collected during scientific trawling operations in Bass Strait, Victoria. This was followed in 1995 [30] with a report on lamellarins Q–R (**11**–**12**) from a second specimen of *Dendrilla cactos* collected by SCUBA off Durras, New South Wales, and in 2012 [31] by a report on lamellarins O1–O2 (**13**–**14**) from an *Ianthella* sp. (CMB-01245) collected in 1991 during scientific trawling operations in Bass Strait, Victoria. In 2014 [19], we reported on the ability of **9** to reverse efflux mediated (BCRP) drug resistance in cancer cells, prompting widespread interest in the scientific community. In addition to sponges, we reported on new lamellarins from Australian collections of marine tunicates [32,33,34], including from *Didemnum* sp. (CMB-01656) collected in 1994 by SCUBA (−20 m) off Wasp Island, Durras, New South Wales, and *Didemnum* sp. (CMB-02127) collected in 1996 during scientific trawling operations off the Northern Rottnest Shelf, Western Australia. To date, our reports of **9**–**14** remain the only accounts of lamellarins from marine sponges, prompting us to speculate whether our collection contained other lamellarin-producing sponges. The GNPS molecular network shown in Figure 5 revealed a family of nodes clustered around the authentic natural product **13** and the monomethyl ether derivative of lamellarin O (dark green) and analogues in the original CMB-01245 extract (red), as well as new nodes (blue) associated with another *Ianthella* sp. (CMB-01311) also collected in 1991 during scientific trawling operations in Bass Strait, Victoria, Australia. This latter specimen contains nodes with unique molecular weights (molecular formula) that warrant more detailed chemical analysis.

### 3.5. Ircinialactams

In 2010 [20], we reported a series of sesterterpene glycinyl-lactams, including ircinialactams A (**15**) and C (**16**), 8-hydroxyircinialactams A (**17**) and B (**18**), and *ent*-ircinialactam C (**19**) from a selection of sponges, including *Ircinia* sp. (CMB-01064) collected in 1990 during commercial trawling operations in the Great Australian Bight, and *Ircinia* sp. (CMB-03363) and *Psammocinia* sp. (CMB-03321)) collected in 2001 by SCUBA near Port Phillip Heads, Victoria, Australia. In 2018 [35], we went on to report additional sesterterpene glycinyl-lactams including ircinialactams B (**20**) and G (**21**), and 8-hydroxyircinialactams C (**22**) and G (**23**) from a series of sponges, including *Sarcotragus* sp. (CMB-01012) and *Psammocinia* sp. (CMB-01018) collected in 1986 and 1988, respectively, by SCUBA (−15 m) off Durras on the mid-south coast of New South Wales. Importantly, selected ircinialactams exhibited promising isoform selective potentiation of glycine gated chloride channel receptors (GlyRs), a unique pharmacology that could inform development of a new first-in-class treatment for chronic inflammatory pain. The GNPS molecular network shown in Figure 6 revealed a family of nodes clustered around the authentic natural products **15**–**16** (dark green) and analogues in the known ircinialactam producer CMB-01064 (red), as well as potentially new ircinialactams (blue) associated with an unidentified sponge CMB-01017 collected in 1986 by SCUBA (−15 m) off Durras on the mid-south coast of New South Wales. Some 35 years after being collected, CMB-01017 would appear to be worthy of further investigation.

### 3.6. Discorhabdins

In 2000 [21], we reported on the new pyrroloiminoquinone discorhabdin R (**24**) from a *Latrunculia* sp. (CMB-02255) collected in 1997 during deep-sea scientific trawling operations (−540 m) off Prydz Bay, Antarctica, and a *Negombata* sp. (CMB-02644) collected in 1998 by SCUBA (−20 m) off Port Campbell, Victoria, Australia. In 2009 [36,37], we reported on the new (+)-dihydrodiscorhabdin A (**25**), (+)-debromodiscorhabdin A (**26**), (+)-dihydrodiscorhabdin L (**27**), and (+)-discorhabdin X (**28**) from a *Higginsia* sp. (CMB-02720) collected in 1998 by SCUBA (−20 m) off Deal island, Bass Strait, Victoria, and a *Spongosorites* sp. (CMB-02523) collected in 1998 by SCUBA (−15 m)off Port Campbell, Victoria. The GNPS molecular network shown in Figure 7 revealed a family of nodes clustered around the authentic natural product discorhabdin D (**29**) (dark green) and analogues in the known discorhabdin producers CMB-02720 and CMB-02523 (red), as well as potentially new discorhabdins (blue) associated with an unidentified sponge CMB-01879 collected in 1995 during scientific trawling operations (−45 m) in the Great Australian Bight.

### 3.7. Trunculins

In 1987 [22], we reported the first occurrence of a family of norsesterterpene cyclic peroxides bearing an unprecedented carbon skeleton, trunculins A–B (**30**–**31**), and their methyl esters **32**–**33** from *Latrunculia brevis* (CMB-01738) collected by SCUBA (−20 m) off South Durras, New South Wales. This was followed in 1993 [38] with a report of trunculin F (**34**) and its methyl ester **35** from *Latrunculia conulosa* collected by SCUBA (−5 m) off Flinders, Victoria, and in 1998 [39], trunculins G–I (**36**–**38**) from a *Latrunculia* sp. collected in 1995 by SCUBA (−30 m) off a World War II submarine scuttled near Port Phillip Heads, Victoria. Terpene cyclic peroxides are a class of natural product unique to marine sponges, with empirical rules for assignment of peroxide configurations based on NMR chemical shifts proposed by Capon in 1985 [40], allowing for rapid identification of natural stereo diversity. The GNPS molecular network shown in Figure 8 revealed a family of nodes clustered around the authentic natural product trunculin B (**30**) (dark green) and analogues in the known trunculin producer CMB-01738 (red), as well as potentially new trunculins (blue) associated with three unidentified sponges; CMB-01001 and CMB-01723 collected in 1985 and 1995, respectively, by SCUBA (−20 m) off Durras, New South Wales, and CMB-01179 collected in 1991 by SCUBA (−25 m) off Port Phillip Heads, Victoria. The expansive diversity of new trunculin nodes suggests that these latter three sponge samples offer the prospect of an array of new members of this relatively rare structure class.

## 4. Case Studies in the Discovery of Rare and New Natural Product Classes

Analysis of GNPS molecular families in Figure 1 revealed several unique clusters associated with only one producing organism [i.e., *Dysidea* sp. (CMB-01171), *Cacospongia* sp. (CMB-03404), and *Thorectandra choanoides* (CMB-01889)]. Further investigations of these sponges resulted in the isolation of new classes of natural products: dysidealactams and dysidealactones [13], cacolides [14], and thorectandrins [15]. The GNPS analysis also identified another producer of the rare trachycladindoles, curiously from a different taxonomical genera as the original producer [12]. All the case studies are briefly summarised below.

### 4.1. Trachycladindoles

In 2008 [26], we reported the first natural examples of an indolo-2-carboxylate bearing a 2-amino-4,5-dihydroimidazole moiety, trachycladindoles A–G (**39**–**45**) from *Trachycladus laevispirulifer* (CMB-03397) collected in 2001 during commercial trawling operations in the Great Australian Bight. We subsequently patented the trachycladindoles as potent and selective kinase inhibitors with potential application in the treatment of neurodegenerative diseases (i.e., Alzheimer’s and Parkinson’s diseases) [41]. Unfortunately, with available supplies limited to <1 mg and targeted resupply of the Australian deep-water source *Trachycladus* sponge impractical, combined with no other literature reports of trachycladindoles, further investigations of this promising pharmacophore were stalled. Efforts at investigating structure activity relationships using available material were also compromised by the observation that on storage (−30 °C in DMSO) trachycladindoles E (**43**) and F (**44**) underwent quantitative deformylation to trachycladindoles A (**39**) and C (**41**) [42].

**Figure 9:**
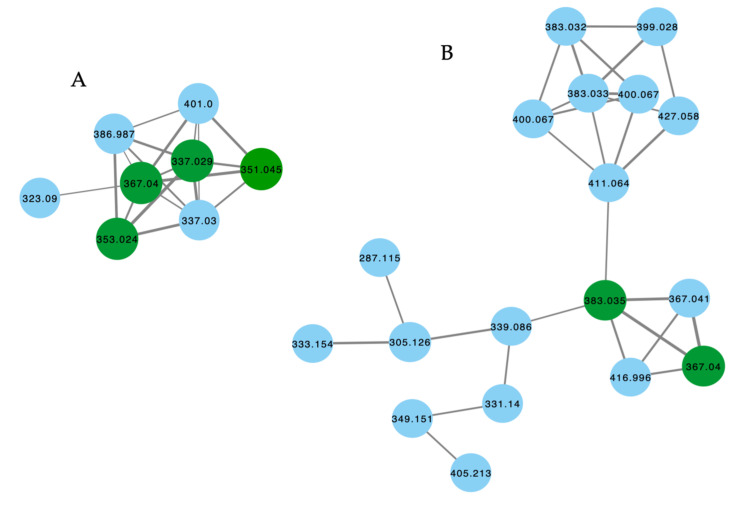

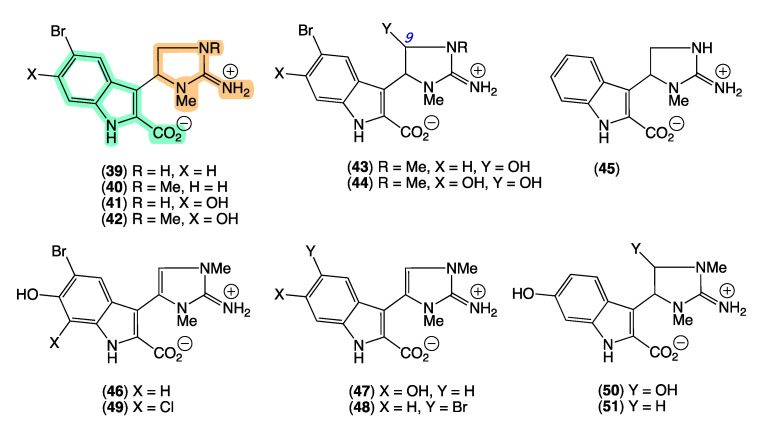
GNPS molecular family and structures for trachycladindoles. Highlights: authentic samples of **39**–**41** and **43** (cluster **A**, dark green); **42** and **44** (cluster **B**, dark green) and putative new trachycladindoles in CMB-01063 (blue), and the indolo-2-carboxylate and 2-amino-4,5-dihydroimidazole sub-structures in common across all trachycladindoles (light green and tan).

The GNPS molecular network shown in Figure 9 revealed two families of nodes: Family A clustered around authentic samples of trachycladindoles A–C (**39–41**) and E (**43**) (dark green), and Family B clustered around authentic samples of trachycladindoles D (**42**) and F (**44**) (dark green), with both families featuring additional nodes (blue) attributed to new trachycladindoles associated with a *Geodia* sp. (CMB-01063) collected in 1990 during commercial trawling operations in the Great Australian Bight. Encouraged by this discovery, in 2020 [12], we reported on the re-isolation of trachycladindoles A–G (**39**–**44**) and an array of new trachycladindoles H–M (**46**–**51**) from CMB-01063. As an interesting aside, the discovery of trachycladindoles in two taxonomically unrelated sponge genera (*Trachycladus* and *Geodia*), and only from Great Australian Bight specimens of these sponges, does fuel speculation that the trachycladindoles may not be sponge natural products but may be microbial in origin.

### 4.2. Dysidealactams

Australian sponges of the genus *Dysidea* have a history of producing structurally diverse natural products, many featuring novel chemical scaffolds and functionality. For example, as early as the 1970s, *Dysidea* specimens collected off the Great Barrier Reef near Townsville and Cooktown, Queensland, were reported to yield brominated diphenyl ethers [43,44] and the chlorinated dysidin and dysidenin [45,46], while specimens collected further south off Gladstone yielded sesquiterpenes spirodysin and trichloroleucine diketopiperazines [47], and those off Cronulla, New South Wales, yielded sesquiterpene furans such as furodysin and furodysinin [48]. Since the 1970s, many other natural products have been reported from Australian *Dysidea*, including sesquiterpenes [49,50,51,52,53], furanosesquiterpenes [54], thiosesquiterpenes [55], chlorinated *N*-acyl amino esters [56], sesterterpene tetronic acids [57], mycosporines [58], chloroleucines [59,60,61], brominated diphenyl ethers [53,62,63,64], dioxanes [65], 9,11-secosterols [66], and polyoxygenated sterols [67,68]. Given this historic productivity, it is perhaps understandable that there has been a decline in the discovery of new classes of natural products from Australian *Dysidea*.

**Figure 10:**
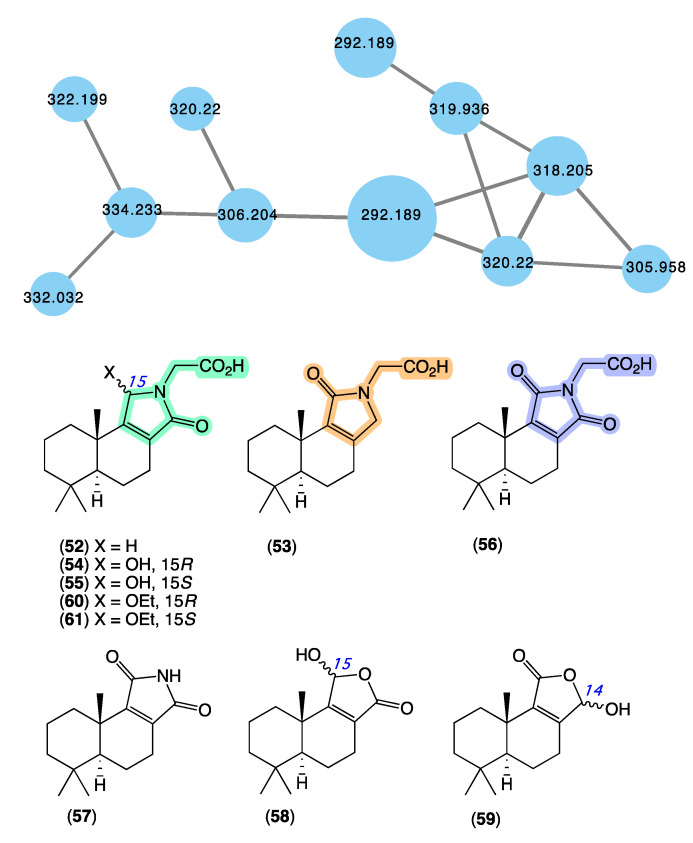
GNPS molecular family (blue) and structures for dysidealactams and dysidealactones. Highlights: the regioisomeric glycinyl-lactam and glycinyl-imide sub-structures (green, tan and purple).

The GNPS molecular network shown in Figure 10 revealed a molecular family uniquely associated with *Dysidea* sp. (CMB-01171) collected in 1991 by SCUBA (−25 m) off Port Phillip Heads, Victoria. Although this specimen had been in our collection for over 30 years, it had failed to be noticed. Consequently, we were intrigued to have the GNPS analysis reveal natural product nodes unique to this *Dysidea* sp., especially given the extensive history of Australian *Dysidea* sp. chemistry (see above). Following a chemical investigation, in 2020 [13], we reported on a suite of new sesquiterpenes from *Dysidea* sp. (CMB-01171), including dysidealactams A–F (**52**–**57**), dysidealactones A–B (**58**–**59**), and two solvolysis artifacts **60**–**61**. Of particular note, dysidealactams A–D (**52**–**55**) incorporate a rare glycinyl lactam moiety (see ircinialactams above), and **56** incorporates an unprecedented glycinyl imide moiety. Due to a prolonged storage in EtOH, it is quite common to observe the esterifaction of carboxylic acid groups to the corresponding ethyl esters [42]. The dysidealactams and dysidealactones immediately attracted the attention of synthetic chemists, with successful synthesis reported in 2023 [69].

### 4.3. Cacolides

**Figure 11:**
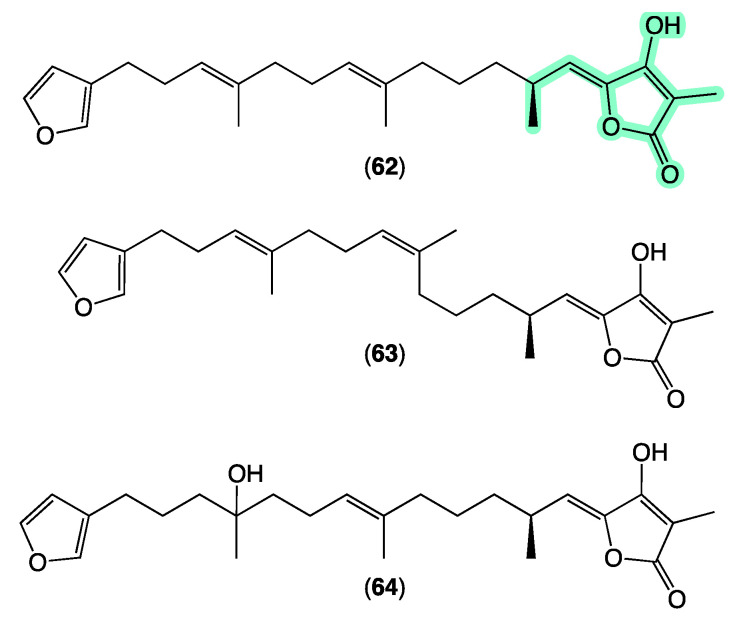
Selected known sesterterpene tetronic acids. Highlights: tetronic acid termini common to all sesterterpene tetronic acids (light green).

Sesterterpene tetronic acids are a family of natural products unique to selected genera of marine sponges, with numerous examples reported over the last 50 years. The earliest accounts, for example, include a 1972 [70] report of ircinin-1 and ircinin-2 from *Ircinia oros* followed shortly after in 1973 [71] by variabilin from *Ircinia variabilis*. Following re-isolation from Australian *Ircinia* spp., absolute configurations were assigned to all three of these earliest exemplars in 1994 [72]. The last four decades of marine natural products research saw many reports of sesterterpene tetronic acids from Irciniidae sponges, including from Australian *Ircinia* [20,73], *Psammocinia* [20,35,74,75,76], and *Sarcotragus* spp. [71]. As the number of known tetronic acids increased, so did the incidence of re-isolation, driving the need for rapid, reliable, and cost-effective methods for dereplication, with many researchers becoming adept at detecting and deprioritizing sesterterpene tetronic acids in crude sponge extracts. Some tell-tales included the observation that extracts/fractions rich in sesterterpene tetronic acids exhibited significant antibacterial activity and diagnostic ^1^H NMR resonances, particularly for the furan and tetronic acid termini. As our experience with the ircinialactams revealed (described above), there is still scope for discovery within the sesterterpene tetronic acid motif. Hence, we were keen to determine if a GNPS molecular network approach could provide a more reliable means of dereplication. To this end, we interrogated our GNPS molecular network of 960 marine extracts using authentic standards of (7*E*,12*E*,20*Z*,18*S*)-variabilin (**62**) (Figure 11) and ircinialactam A (**15**) (Figure 6). Unsurprisingly for such a common structure class, a great many marine extracts co-clustered with the authentic standards. To better understand these data, we elected to further refine the visualization into three separate analyses based around specimens belonging to each of the genera *Ircinia*, *Psammocinia* and *Sarcotragus*.

**Figure 12:**
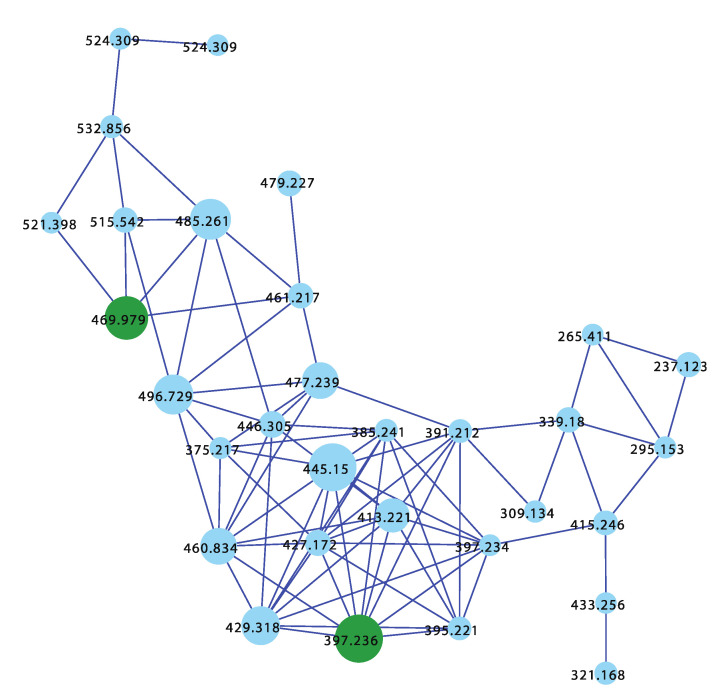
GNPS molecular family for *Ircinia* spp. CMB-01064, CMB-03363, CMB-01058, CMB-01693, and CMB-02014. Highlights: authentic standards for **62** and **15** (dark green), sesterterpene tetronic acids common to CMB-01064, CMB-03363, CMB-01058, CMB-01693, and CMB-02014 (blue).

The sponge *Ircinia* sp. (CMB-01064) collected in 1990 during commercial trawling operations in the Great Australian Bight was known to produce (7*E*,12*E*,20*Z*,18*S*)-variabilin (**62**), (7*E*,12*Z*,20*Z*,18*S*)-variabilin (**63**), (12*E*,20*Z*,18*S*)-8-hydroxy-variabilin (**64**), ircinialactam A (**15**), and 8-hydroxyircinialactams A (**17**) and B (**18**) (Figure 6 and Figure 11); while *Ircinia* sp. (CMB-03363) collected in 2001 by SCUBA near Port Phillip Heads, Victoria, was known to produce **62**, **63**, **64**, and **15**. Three additional *Ircinia* spp CMB-01058 were collected in 1990 by SCUBA near Flinders, Victoria; CMB-01693 collected in 1994 by benthic grab (−71 m) during a scientific operations off Port Lincoln, South Australia, and CMB-02014 collected in 1995 by SCUBA in Port Phillip Bay, Victoria, had not been previously subjected to chemical analysis. The GNPS molecular network shown in Figure 12 on these five *Ircinia* spp. revealed a common molecular family co-clustering with authentic samples of **62** and **15** (dark green)—confirming that GNPS could be used to detect/dereplicate *Ircinia* sesterterpene tetronic acids.

**Figure 13:**
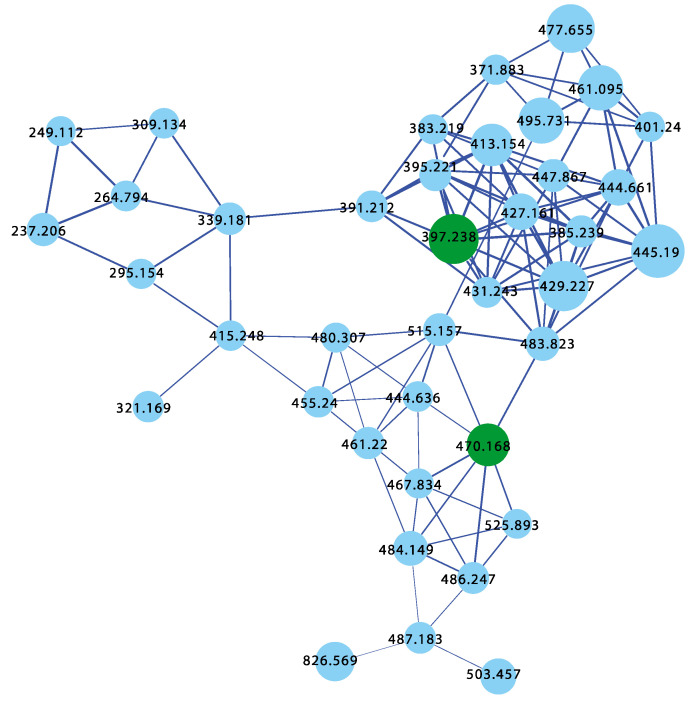
GNPS molecular family for *Psammocinia* spp. CMB-03231, CMB-01018, CMB-03344, CMB-01757, and CMB-02026. Highlights: authentic standards for **62** and **15** (dark green), sesterterpene tetronic acids common to CMB-03231, CMB-01018, CMB-03344, CMB-01757, and CMB-02026 (blue).

The sponges *Psammocinia* sp. (CMB-03231) collected in 2000 by SCUBA off Lonsdale, Victoria, *Psammocinia* sp. (CMB-01018) collected in 1988 by SCUBA off Durras, New South Wales, and *Psammocinia* sp. (CMB-03344) collected in 2001 by SCUBA off Port Phillip Heads, Victoria, were all known to produce sesterterpene teronic acids; while *Psammocinia* sp. (CMB-01757) collected in 1995 by SCUBA (−30 m) off Barwon Heads, Victoria, and *Psammocinia* sp. (CMB-02026) collected in 1995 during scientific trawling operations (−100 m) in the Great Australian Bight, was not subjected to chemical analysis. The GNPS molecular network shown in Figure 13 on these five *Psammocinia* spp. revealed a common molecular family co-clustering with authentic samples of **62** and **15** (dark green)—confirming that GNPS could be used to detect/dereplicate *Psammocinia* sesterterpene tetronic acids.

**Figure 14:**
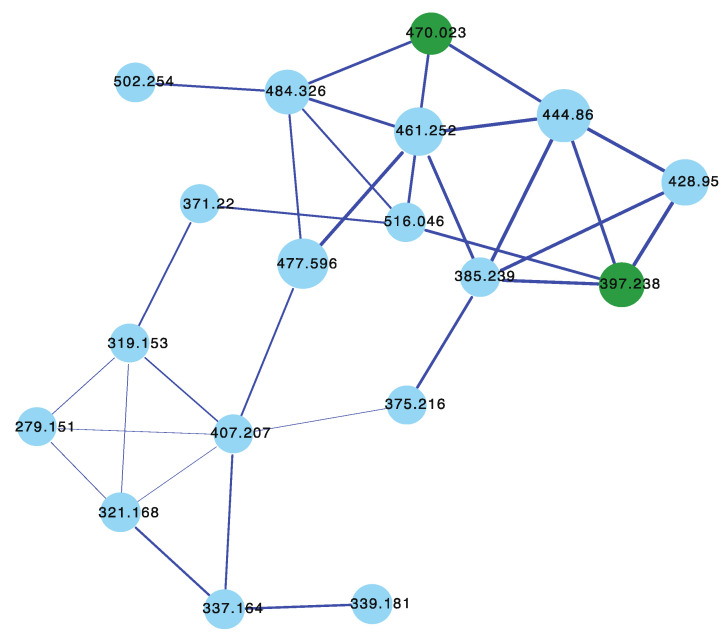
GNPS molecular family for *Sarcotragus* spp. CMB-01788, CMB-01848, CMB-02707, CMB-02717, and CMB-03390. Highlights: authentic standards for **62** and **15** (dark green), sesterterpene tetronic acids common to CMB-01788, CMB-01848, CMB-02707, CMB-02717, and CMB-03390 (blue).

The sponges *Sarcotragus* sp. (CMB-01788) and *Sarcotragus* sp. (CMB-01848) collected in 1995 during scientific trawling operations (−30 m and −85 m, respectively) in the Great Australian Bight, *Sarcotragus* sp. (CMB-02707) and *Sarcotragus* sp. (CMB-02717) collected in 1998 by SCUBA (−10 m) off Deal Island, Bass Strait, and *Sarcotragus* sp. (CMB-03390) collected in 2001 during commercial trawling operations in the Great Australian Bight, had not been subjected to chemical analysis. The GNPS molecular network shown in Figure 14 on these five *Sarcotragus* spp. revealed a common molecular family co-clustering with authentic samples of **62** and **15** (dark green)—confirming that GNPS could be used to detect/dereplicate *Sarcotragus* sesterterpene tetronic acids.

**Figure 15:**
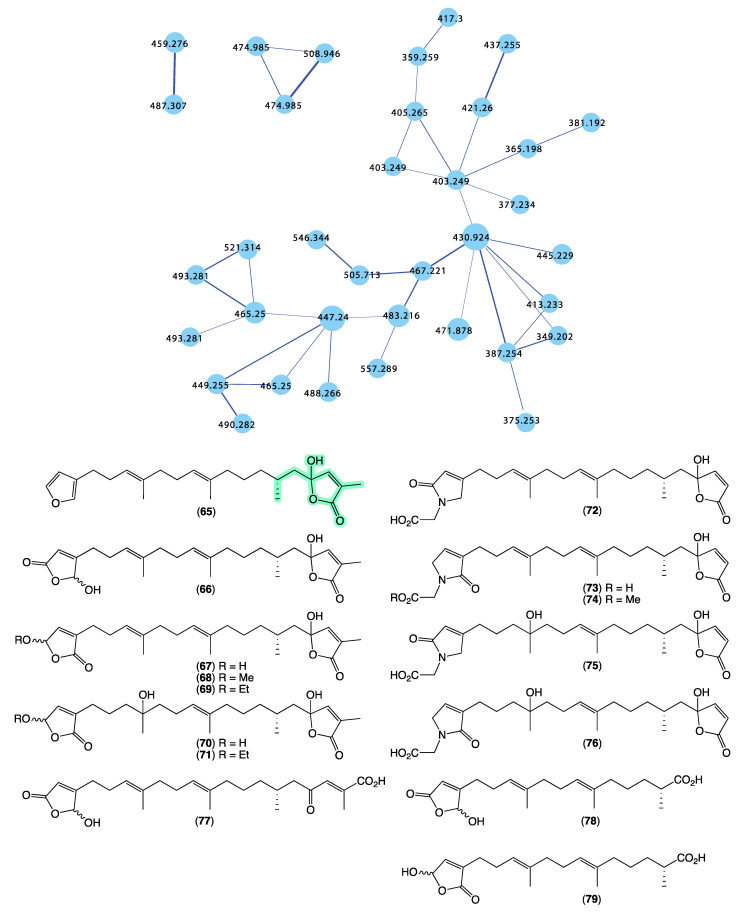
GNPS molecular family and structures for cacolides A–L (**65**–**76**) and cacolic acids A–C (**77**–**79**). Highlights: cacolides and cacolic acids in CMB-03404 (blue), and the unique α-methyl-γ-hydroxybutenolide sub-structures in common across many cacolides (light green).

Returning to the GNPS molecular network, we noted that *Cacospongia* sp. (CMB-03404) collected in 2001 during commercial trawling operations in the Great Australian Bight featured a unique molecular family (Figure 15) that did not share any nodes with authentic sesterterpene tetronic acids, or any of the *Ircinia*, *Psammocinia*, or *Sarcotragus* spp. extracts noted above. This was initially perplexing as at an earlier time, and using more traditional UPLC-DAD and ^1^H NMR dereplication approaches, we dismissed this extract as containing known sesterterpene tetronic acids. Clearly, our initial assessment was incorrect, with the GNPS analysis prompting a more detailed chemical investigation. In 2018 [14], we reported on an unprecedented family of sesterterpene α-methyl-γ-hydroxybutenolides, cacolides A–L (**65**–**76**), together with the biosynthetically related cacolic acids A–C (**77**–**79**) from *Cacospongia* sp. (CMB-03404) (Figure 15). Had it not been for the detection of a unique GNPS molecular family for CMB-03404, it is likely that our original dereplication assessment would had held, and this extract would have remained dormant in our collection for many more years. This experience reinforces the need to be cautious in all dereplication, and be willing to revise determinations when contradictory data emerge.

### 4.4. Thorectandrins

The bright yellow tryptophan alkaloid aplysinopsin (**80**), first reported in 1977 [77] from a Great Australian Bight sponge of the genus *Thorecta* sp., heralded the discovery of a great many related and uniquely sponge-derived natural products, including from the genera *Verongia*, *Dercitus*, *Smenospongia*, *Dictyoceratida*, *Aplysina*, *Hyrtios*, and *Thorectandra* [78]. Many examples of the aplysinopsin family of natural products were reported to exhibit prominent biological properties ranging across antimicrobial, antiviral, antimalarial, anticancer, anti-cholinesterase, and anti-depressant activity [79]. Indeed, some 36 years after first being reported from a northern Australian sponge, we returned to the aplysinopsin scaffold and, in 2013 [80], reported on a series of new analogues from the southern Australia sponge *Ianthella* cf. *flabelliformis* (CMB-03322)—examples of which exhibited promising isoform selective GlyRs potentiation with possible application in the development of a new class of analgesic for the treatment of chronic inflammatory pain (see ircinialactams above). Nevertheless, with such an extensive literature on aplysinopsins, the prospect of new discoveries seemed slight—that is, until we reviewed our GNPS data.

**Figure 16:**
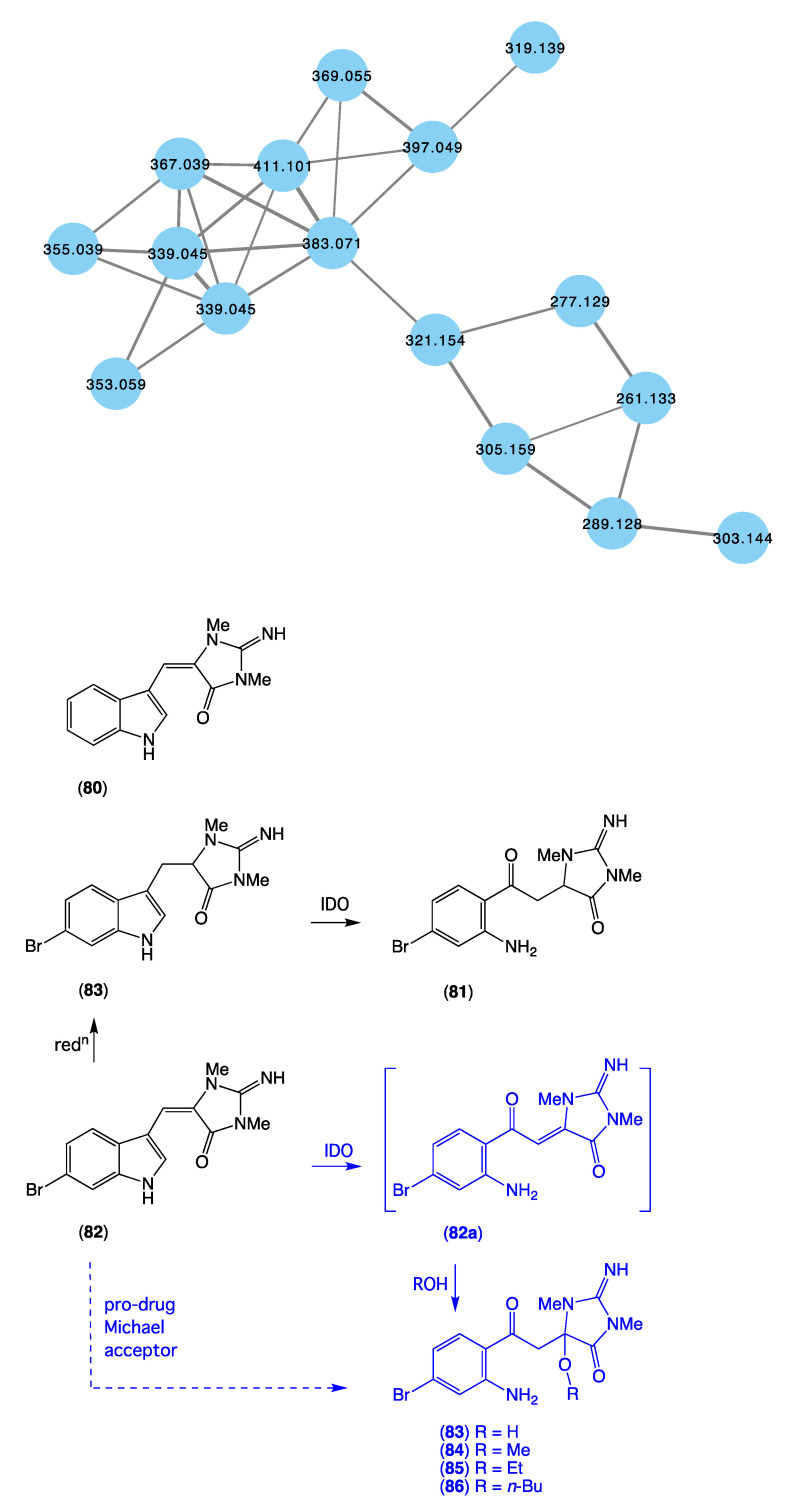
GNPS molecular cluster uniquely associated with *Thorectandra choanoides* (CMB-01889) (blue), and structures of aplysinopsin (**8**), and selected CMB-01889 metabolites **81**–**83** and solvolysis artifacts **83**–**86**.

The GNPS molecular network shown in Figure 16 revealed a unique molecular family inclusive of multiple nodes associated with *Thorectandra choanoides* (CMB-01889) collected in 1995 during deep water (−45 m) scientific trawling operations in the Great Australian Bight. As a result, this sponge was targeted for detailed chemical analysis and, in 2021 [15], we reported on a new tryptophan alkaloid, thorectandrin A (**81**), along with an array of known analogues, including 6-bromoaplysinopsin (**82**), 6-bromo-1′,8-dihydroaplysinospin (**83**), and an array of solvolysis adducts **84**–**87**. The GNPS thorectandrin molecular family clusters were particularly useful at revealing solvolysis artifacts. Close examination of these compounds led us to speculate that **82** (and **80**) could serve as a substrate for an indoleamine 2,3-dioxygenase-like (IDO) enzyme, resulting in transformation to the intermediate ring opened produce **82a**, which, as a potent Michael acceptor, could act as a cellular toxin (explaining some of the observed “aplysinopsin” biology). Alternatively, the intermediate Michael acceptor **82a** could form solvolysis adducts during handling, such as **83**–**86**. By contrast, the reduced aplysinopsin analogue **83** might also be viewed as a prospective IDO substrate, being transformed to the thorectandin **81** which was not a Michael acceptor and, hence, not toxic. As a result, the discovery of **81** shed light on a previously unappreciated “aplysinopsin” chemical reactivity and ecology, where some aplysinopsins (i.e., **80** and **82**) can be viewed as pro-drug Michael acceptors that are activated by IDO’s in the tissues of species that prey on sponges, allowing them to act as potent Michael acceptor toxins. This same mechanism of action likely plays out in mammalian and microbial cells, explaining the anticancer and anti-infective properties of selected aplysinopsins that are pro-drug Michael acceptors.

## 5. Conclusions

Hopefully the case studies summarized above revealed how GNPS molecular networking can extract new value from a library of 960 southern Australian marine extracts including;

- Disclosing the rarity of a structure class (i.e., dragmacidins);
- Finding new sources/examples of rare chemistry (i.e., trachycladindoles);
- Prioritizing otherwise stranded extracts to help find new analogues of known structure classes (i.e., franklinolides, bistellettazines, lamellarins, ircinialactams, discorhabdins, trunculins);
- Finding entirely new structure classes (i.e., dysidealactams, cacolides); and
- Discovering new natural products that shed light on ecological properties and pharmacological mechanisms of action (i.e., thorectandrins).

In conclusion, we would like to share observations on some challenges/limitations of GNPS molecular networking:

- *Access to UPLC-MS/MS technology*: While UPLC-MS/MS technology is increasingly accessible, the levels of access (and cost) can vary greatly between laboratories. To best apply GNPS as a routine biodiscovery dereplication tool requires hands on and reliable access to appropriate instrumentation.
- *Access to HRESIMS*: In our hands, the accuracy of the *m/z* values acquired for each GNPS node was insufficient to assign definitive molecular formula (MF). As MF assignments are critical to online database searches (i.e., SciFinder) that are the key to rapidly discriminating known from new natural products, this necessitates separate measurements on targeted molecular families and individual nodes. In due course this technological limitation may be solved by better instrumentation.
- *Clusters versus singletons*: In any GNPS molecular network, what stands out most are the molecular families (i.e., the clusters of nodes linked by solid lines of varying thickness). That said, all GNPS molecular networks feature a large number of singletons—nodes that are not clustered. While it may be tempting to disregard singletons during any GNPS analysis, this runs the risk of overlooking interesting (even remarkable) natural products—that simply are so unique in their MS/MS fragment that they have no cluster partners. Singletons can (on occasion) count!
- *Molecular families*: Molecular families are clusters of natural products (nodes) that share a common/related MS/MS fragmentation. As such, members of any given structure class can be incorporated into one or more molecular families, depending on the nature of structure diversity and its impact on MS/MS fragmentation. Do not assume all members of the same structure class will co-cluster.
- *Molecular adducts*: Some natural products classes can exist as multiple adducts (i.e., M+H, M+Na, M+K, M+NH_4_), which can cluster separately into different molecular families. This can create the illusion of more structure diversity than is actually the case.
- *Ionization*: Some natural products do not ionize well under ESI(+) conditions, and as such, would not feature prominently (or at all) in a +ve mode GNPS molecular network. Depending on the structure class you are looking for, you might consider reacquiring a –ve mode GNPS molecular network.
- *Structure Proof*: Finally, and arguably of greatest importance, there are occurrences in the literature where researchers seemed to defer to GNPS as a definitive proof of structure assignment. This is unwise, especially for chiral natural products, as MS/MS fragmentation is silent on matters of stereochemistry. GNPS can be a valuable guide for dereplication and prioritization, but unless you have access to an authentic natural product standard, it is not a substitute for isolation and more traditional structure elucidation based on spectroscopic analysis.

## Figures and Tables

**Table 1:** Marine sponges known to producing different classes of natural product.

Specimens Known to Produce Chemistry	Compound Classes [Ref]
*Geodia* sp. (CMB-01989)	franklinolides [16]
*Stelletta* sp. (CMB-01936)	bistellettazines [17]
*Leiosella/Halichondria* spp. (CMB-02782)	dragmacidins [18]
*Ianthella* sp. (CMB-01245)	lamellarins [19]
*Ircinia* sp. (CMB-01064)	ircinialactams [20]
*Latrunculia* sp. (CMB-02720)	discorhabdins [21]
*Spongosorites* sp. (CMB-02523)	discorhabdins [21]
*Latrunculia* sp. (CMB-01738)	trunculins [22]
*Phorbas* sp. (CMB-01978)	phorbasins [23]
*Phorbas* sp. (CMB-01934)	phorbasins [23]
*Phorbas* sp. (CMB-02020)	phorbasins [23]
*Spongia* sp. (CMB-03399)	heterofibrins [24]
*Amphimedon* sp. (CMB-01607)	amphilactams [25]
*Amphimedon* sp. (CMB-01871)	amphilactams [25]
*Amphimedon* sp. (CMB-01876)	amphilactams [25]
*Trachycladus* sp. (CMB-03397)	trachycladindoles [26]

**Table 2:** Taxonomically identified sponges (409/960 specimens).

Genus	#	Genus	#	Genus	#
*Acanthella*	3	*Erylus*	1	*Phorbas*	2
*Acarnus*	3	*Fasciospongia*	3	*Phoriospongia*	28
*Amphimedon*	2	*Gelliodes*	1	*Phyllospongia*	2
*Ancorina*	5	*Geodia*	6	*Polymastia*	4
*Anomoianthella*	1	*Grantia*	1	*Psammastra*	1
*Aplysina*	5	*Guitarra*	1	*Psammocinia*	11
*Arenochalina*	2	*Halichondria*	13	*Psammoclemma*	5
*Axinella*	13	*Halisarca*	1	*Pseudoceratina*	1
*Biemna*	2	*Hemiasterella*	1	*Pseudotrachya*	1
*Cacospongia*	2	*Hemigellius*	1	*Ptilocaulis*	3
*Callyspongia*	9	*Higginsia*	1	*Raphidotethya*	3
*Carteriospongia*	1	*Holopsamma*	6	*Raphoxya*	1
*Caulerpa*	1	*Homaxinella*	2	*Raspailia*	3
*Chelonaplysilla*	1	*Hyattella*	2	*Reniera*	1
*Chondropsis*	4	*Hymeniacidon*	2	*Rossella*	1
*Cinachyra*	2	*Iotrochola*	1	*Sarcotragus*	2
*Clathria*	20	*Ianthella*	4	*Sigmaxinella*	2
*Cliona*	1	*Ircinia*	3	*Siphonochalina*	1
*Coscinoderma*	2	*Isodictya*	2	*Spirastrella*	37
*Crella*	2	*Latrunculia*	2	*Spongia*	7
*Cribrochalina*	18	*Leiosella*	7	*Spongionella*	3
*Cymbastela*	6	*Lendenfeldia*	2	*Spongosorites*	3
*Dactylia*	2	*Leucetta*	3	*Stelletinopsis*	4
*Darwinella*	1	*Luffariella*	2	*Stelletta*	2
*Dendrilla*	1	*Melonanchora*	1	*Strongylacidon*	3
*Dendrilla*	1	*Microxina*	1	*Strongylodesma*	1
*Desmacidon*	1	*Mycale*	4	*Stylotricophora*	2
*Desmapsamma*	2	*Myrmekioderma*	1	*Suberites*	4
*Dictyodendrilla*	1	*Myxilla*	5	*Taunura*	3
*Dictyosphaeria*	1	*Neofibularia*	1	*Tedania*	1
*Druinella*	1	*Niphates*	1	*Tedaniosis*	1
*Dysidea*	1	*Oceanapia*	9	*Tethya*	6
*Echinochalina*	4	*Oxymycale*	5	*Tetilla*	4
*Echinoclathria*	4	*Paracornulum*	1	*Thorecta*	3
*Echinodictyum*	8	*Parahigginsia*	2	*Thorectandra*	7
*Ecionemia*	1	*Pericharax*	2	*Trachycladus*	5
*Ectyomyxilla*	1	*Phakellia*	3	*Xestospongia*	1
*Ectyoplasia*	1				

# = number of samples belonging to each genera.

## Data Availability

The full MS/MS data of the 980 sponge extracts can be accessed from ftp://massive.ucsd.edu/MSV000086621/ (accessed on 16 July 2023).

## Supplementary Materials

The following supporting information can be downloaded at: https://www.mdpi.com/article/10.3390/md21070413/s1, Figure S1: GNPS molecular network for 960 marine extracts (high resolution pdf file).
