# Supplementary figures and images for: Case Studies in Molecular Network-Guided Marine Biodiscovery
Source: Mar Drugs. 2023 Jul 20;21(7):413. doi: 10.3390/md21070413 (PMC10381900; doi:10.3390/md21070413)

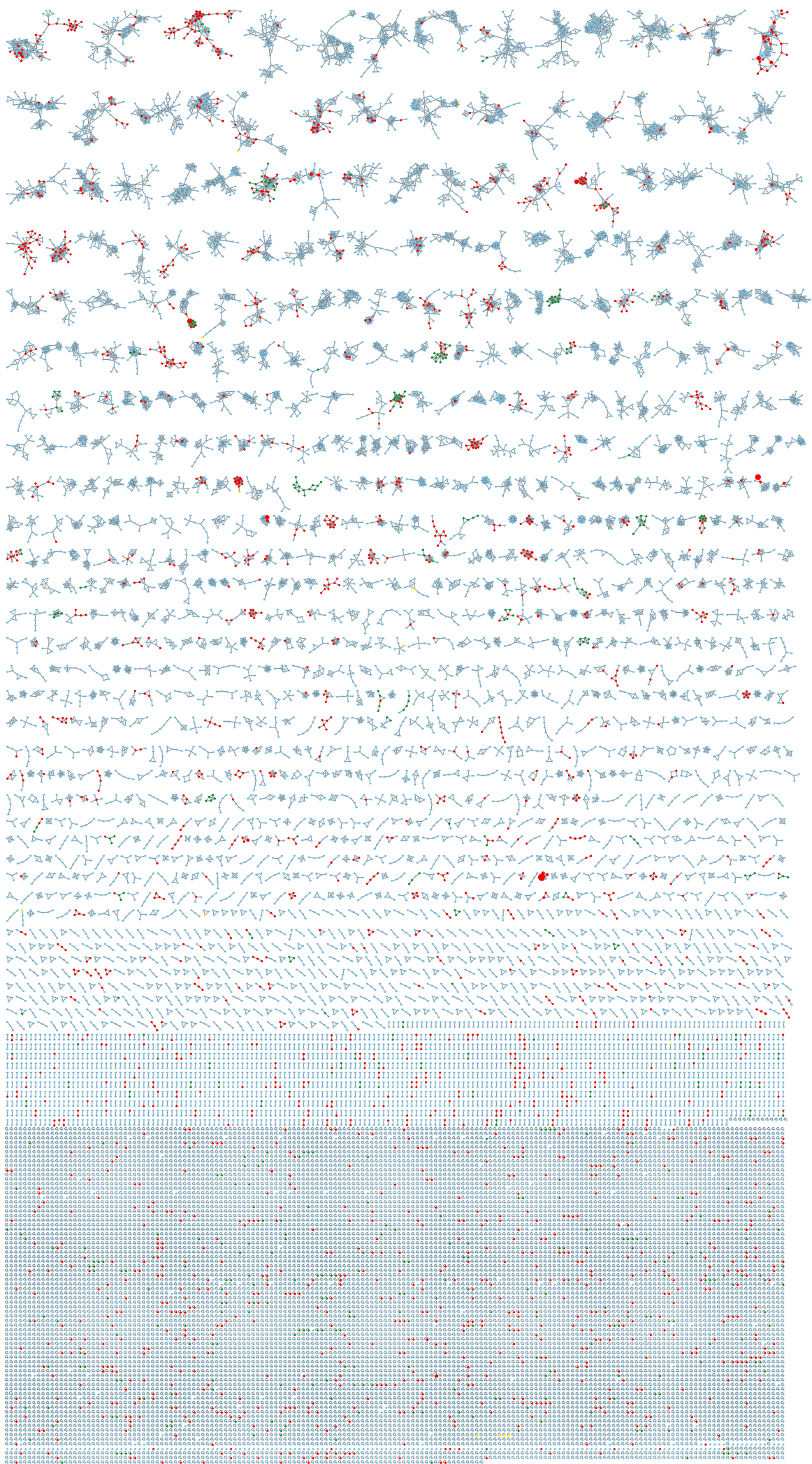

Supplement: Supplementary file 1 [file marinedrugs-21-00413-s001.zip › marinedrugs-2491652-Figure S1.pdf]
